# Age of Synchronization Minimization Algorithms in Wireless Networks with Random Updates under Throughput Constraints †

**DOI:** 10.3390/e25091331

**Published:** 2023-09-14

**Authors:** Yuqiao He, Guozhi Chen, Yuchao Chen, Jintao Wang, Jian Song

**Affiliations:** 1 Beijing National Research Center for Information Science and Technology (BNRist), Beijing 100084, China; heyq18@mails.tsinghua.edu.cn (Y.H.); chen-gz19@mails.tsinghua.edu.cn (G.C.); cyc20@mails.tsinghua.edu.cn (Y.C.); wangjintao@tsinghua.edu.cn (J.W.); 2 Department of Electronic Engineering, Tsinghua University, Beijing 100084, China; 3 Research Institute, Tsinghua University in Shenzhen, Shenzhen 518057, China; 4 Shenzhen International Graduate School, Tsinghua University, Shenzhen 518055, China

**Keywords:** age of synchronization, scheduling, throughput, wireless network

## Abstract

This study considers a wireless network where multiple nodes transmit status updates to a base station (BS) through a shared bandwidth-limited channel. Considering the random arrival of status updates, we measure the data freshness with the age of synchronization (AoS) metric; specifically, we use the time elapsed since the latest synchronization as a metric. The objective of this study is to minimize the weighted sum of the average AoS of the entire network while meeting the minimum throughput requirement of each node. We consider both the central scheduling scenario and the distributed scheduling scenario. In the central scheduling scenario, we propose the optimal stationary randomized policy when the transmission feedback is unavailable and the max-weight policy when it is available. In the distributed scenario, we propose a distributed policy. The complexity of the three scheduling policies is significantly low. Numerical simulations show that the policies can satisfy the throughput constraint in the central controlling scenario and the AoS performance of the max-weight policy is close to the lower bound.

## 1. Introduction

**Figure 1:**
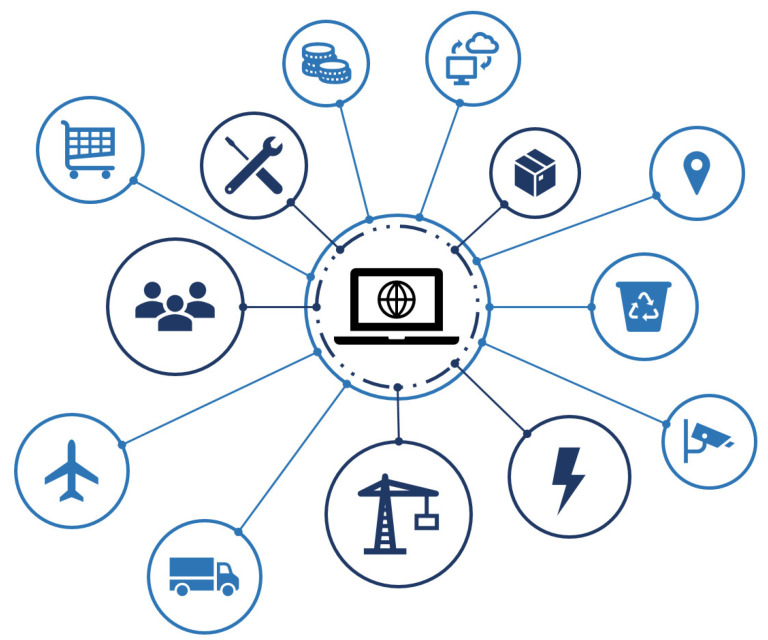
An industrial IoT network contains many components.

With the development of various time-sensitive applications, such as the industrial Internet of Things and automatic driving, the freshness of information has attracted more and more attention. It is important to transmit time-sensitive information to the central controller in a timely manner in order to ensure safety and efficient control in these networks. Figure 1 shows a scenario for the industrial IoT where the central processor monitors the real-time status of various network components, including materials, equipment, power, transportation, staff, etc.

To evaluate the freshness of status updates, researchers have introduced the concept of the age of information (AoI) [1], which is defined as the time elapsed since the the generation of the most recent status update. Previous studies [2,3,4,5,6,7,8,9,10] have focused on developing scheduling algorithms that minimize the average AoI across the network in the generate-at-will scenario, which means the generation of status update packets can be controlled. Various low-complexity algorithms were presented in [2] to minimize the average AoI in the network, demonstrating their proximity to the theoretical lower bound. Power constraints and cross-layer scheduling were considered in [6], where the authors formulated the AoI optimization problem as a constrained Markov decision process and proposed an effective asymptotic optimal scheduling algorithm.

In addition to the generate-at-will scenario, researchers also focus on the random-update scenario, where status updates arrive at terminal devices randomly. Several scheduling algorithms have been proposed in [11,12,13,14,15,16] to minimize the average AoI. However, in the random-update scenario, the AoI may not show the desynchronization of the information. For example, if no new update arrives after the latest update is received, the information at the receiver remains fresh while the AoI still keeps increasing.

Therefore, the concept of the age of synchronization (AoS) was proposed in [17], which is defined as the time elapsed since the desynchronization of the freshest information at the receiver. An age-based max-weight policy was proposed in [18] to minimize the time-average AoS in error-free transmission scenarios. In [19], scheduling algorithms based on the Markov decision process and the restless multi-arm bandit were designed to optimize AoS in unreliable wireless transmission channels. Additionally, when transmitting over unreliable time-varying wireless channels, the authors in [20] modeled the AoS optimization problem as a constrained Markov decision process and revealed a threshold structure for the optimal policy. The primary objective of these works was to minimize the time-average AoS of the whole system. However, in broadcast services, network users may possess varying levels of importance and diverse requirements, such as different throughput needs. Previous studies have investigated the design of scheduling strategies that optimize data freshness, taking into account heterogeneous requirements such as timely throughput and transmission delay [5,21,22,23]. These works indicated that the AoS optimization strategies may cause unfairness among different users.

To address this issue, we consider a model in which each user in the network has a time-average throughput need. Our objective is to propose scheduling policies that not only minimize the time-average AoS for network users but also ensure that the throughput requirement of each user is met. Under different conditions, we propose three corresponding scheduling policies. To be specific, we propose the optimal stationary randomized policy, the max-weight policy, and the distributed policy to deal with ACK-available/ACK-unavailable and centralized/distributed scenarios, respectively. Part of this work has been published in Proceedings of the 2022 IEEE International Symposium on Broadband Multimedia Systems and Broadcasting (BMSB) [24].

The rest of the paper is structured in the following manner. Section 2 introduces the system model, the constraints, and the definition of AoS, and the optimization problem is modeled. In Section 3, a lower bound of the AoS optimization problem is presented. In Section 4, we proposed three scheduling policies—the optimal stationary randomized policy, the max-weight policy, and the distributed policy—and give the corresponding algorithms. In Section 5, the performance of the policies is shown and compared with numerical simulations. Finally, Section 6 concludes the paper.

## 2. System Model

**Figure 2:**
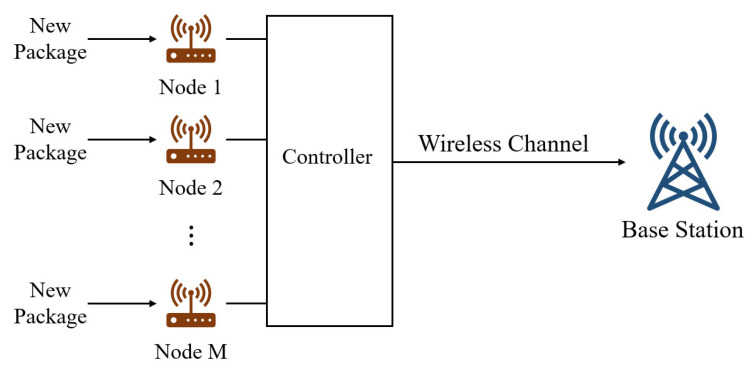
System model.

We consider a model where *M* nodes transmit random status updates to a base station (BS), as illustrated in Figure 2. The time is slotted and denoted by k∈{1,2,…,K}. An indicator function $\Lambda_{i}\left(k\right)$ records whether a new update arrives at node i∈{1,2,…,M} in the time slot before time slot *k*. $\Lambda_{i}\left(k\right)$ follows an independent and identical Bernoulli distribution with parameter $\lambda_{i}$.

The nodes transmit status updates the BS through a shared channel. $u_{i}\left(k\right)$ denotes the transmission indicator function, meaning that $u_{i}\left(k\right)=1$ suggests node *i* transmits an update to the BS at the beginning of time slot *k*. The transmission from node *i* succeeds with probability $p_{i}$. An indicator of successful transmission is denoted by $d_{i}\left(k\right)$. If $d_{i}\left(k\right)=1$, the transmission succeeds and the BS receives the update from node *i* by the end of time slot *k*.

If $u_{i}\left(k\right)=1$ at time slot *k*, node *i* transmits an update to the BS. $d_{i}\left(k\right)=1$ with probability $p_{i}$ and $d_{i}\left(k\right)=0$ with probability $1-p_{i}$. If $u_{i}\left(k\right)=0$, indicating that node *i* does not transmit to the BS in time slot *k*, $d_{i}\left(k\right)$ must be 0.

### 2.1. Age of Synchronization

**Figure 3:**
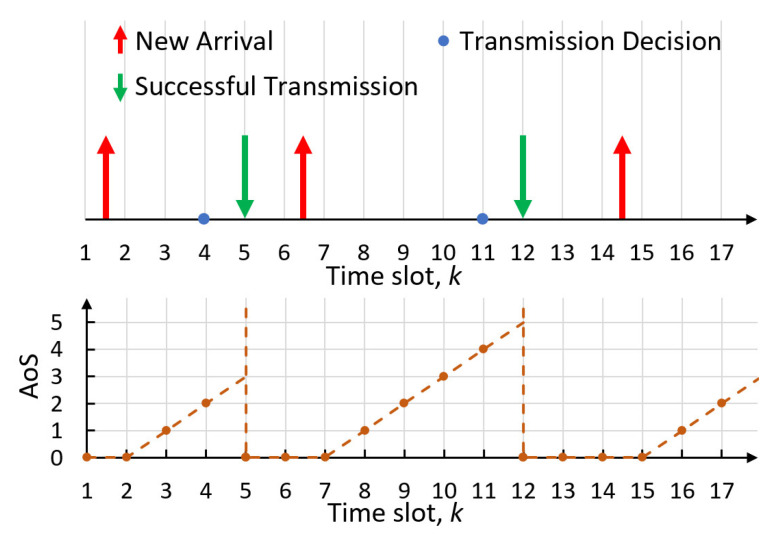
A demonstration of random arrival and update transmission is shown above. The evolution of the AoS is shown below.

The age of synchronization (AoS) describes the time elapsed since the last time that the newest information at the receiver was synchronized with the transmitter. we define $s_{i}\left(k\right)$ as the AoS of node *i* at the end of time slot *k*. The evolution of the AoS with random arrival and successful transmission is illustrated in Figure 3. While the latest update is successfully transmitted in time slot 5, the AoS of node *i* drops to 0 and remains 0 until a new update arrives during time slot 7. Then, the AoS of node *i* keeps increasing by 1 in every subsequent time slot until the new update is received by the BS in time slot 12.

As the BS is only interested in the newest updates, each node always transmits the newest updates in the buffer to the BS. The evolution of $s_{i}\left(k\right)$ follows:

(1) $$s_{i}\left(k\right)=\left\{\begin{array}{cc} 0, & \mathrm{if}\;d_{i}\left(k\right)=1;\\ 0, & \mathrm{if}\;s_{i}\left(k-1\right)=0,\;\mathrm{and}\;\Lambda_{i}\left(k\right)=0;\\ s_{i}\left(k-1\right)+1, & \mathrm{otherwise}. \end{array}\right.$$

We consider that the AoSs of different nodes have different importance, which is represented by $\alpha_{i}>0$, and we are interested in the *expected weighted sum AoS* of the network. We denote the expected weighted-sum AoS during the first *K* time slots under scheduling policy π as

(2) $$J_{K}^{\pi }=\frac{1}{KM}E\left[\sum_{k=1}^{K}\sum_{i=1}^{M}\alpha_{i}s_{i}\left(k\right)|s_{i}\left(0\right)\right],$$

where the expectation is denoted with respect to the randomness of the channel and the scheduling policy.

Without loss of generality, we assume that the sources are synchronized at the beginning, so the initial values of the AoS follow $s_{i}\left(0\right)=0,\;\forall i\in \left\{1,\ldots ,M\right\}$.

### 2.2. Constraints

We consider that the nodes transmit to the BS through a bandwidth-limited channel and the bandwidth requires that one node at most can transmit in each time slot. This *bandwidth constraint* can be formulated as

(3) $$\sum_{i=1}^{M}u_{i}\left(k\right)\leq 1,\;\forall k\in \left\{1,\ldots ,K\right\}.$$

We consider that each node in the network has a minimum throughput requirement and let $q_{i}$ denote the minimum time-average throughput requirement of node *i*. The long-term time-average throughput under a specific policy π denoted by ${\hat{q}}_{i}^{\pi }$ can be computed by

(4) $${\hat{q}}_{i}^{\pi }:=\lim_{K\to \infty }\frac{1}{K}\sum_{k=1}^{K}E_{\pi }\left[d_{i}\left(k\right)\right].$$

Then, the *throughput constraint* can be expressed as

(5) $${\hat{q}}_{i}^{\pi }\geq q_{i},\;\forall i\in \left\{1,\ldots ,M\right\}.$$

The minimum throughput requirement of the entire network is feasible under the following assumption.

**Assumption** **1.**
(6) $$\begin{array}{cc} \sum_{i=1}^{M}\frac{q_{i}}{p_{i}} & \leq 1, \end{array}$$

(7) $$\begin{array}{cc} q_{i} & \leq \lambda_{i},\;\forall i\in \left\{1,\ldots ,M\right\}. \end{array}$$

### 2.3. Optimization Problem

We now formulate the optimization problem as follows.

**Problem** **1** (AoS optimization).

(8a) $$\pi^{*}=\underset{\pi \in \Pi }{\arg\min}\left\{\lim_{K\to \infty }J_{K}^{\pi }\right\},$$

(8b) $$\begin{array}{cc} s.t.\; & {\hat{q}}_{i}^{\pi }\geq q_{i},\;\forall i\in \left\{1,\ldots ,M\right\}; \end{array}$$

(8c) $$\begin{array}{cc} \; & \sum_{i=1}^{M}u_{i}\left(k\right)\leq 1,\;\forall k\in \left\{1,\ldots ,K\right\}. \end{array}$$

Equation (8b) describes the throughput constraints and (8c) describes the bandwidth constraints. We denote the optimal expected weighted-sum AoS under the AoS-optimal policy as

(9) $$AoS^{*}=\lim_{K\to \infty }J_{K}^{\pi^{*}}$$

Then, we give a lower bound of the AoS optimization problem (Equations (8a)–(8c)) first. In three different cases, three scheduling policies are proposed.

## 3. Lower Bound

A lower bound of Equations (8a)–(8c) is obtained as follows.

**Theorem** **1.** *The AoS optimization problem (Equations* (8a)–(8c)) *can be lower bounded by Equations* (10a)–(10c) *for every network setup $\left(M,\lambda_{i},p_{i},q_{i},\alpha_{i}\right)$.*

(10a) $$AoS_{LB}=\min_{{\hat{q}}_{i}}\left\{\frac{1}{2M}\sum_{i=1}^{M}\alpha_{i}\left(\frac{1}{{\hat{q}}_{i}}+\frac{2-2\lambda_{i}}{\lambda_{i}^{2}}{\hat{q}}_{i}-\frac{2-\lambda_{i}}{\lambda_{i}}\right)\right\},$$

(10b) $$\begin{array}{cc} s.t.\; & {\hat{q}}_{i}\geq q_{i},\;\forall i\in \left\{1,\ldots ,M\right\}; \end{array}$$

(10c) $$\begin{array}{cc} \; & \sum_{i=1}^{M}u_{i}\left(k\right)\leq 1,\;\forall k\in \left\{1,\ldots ,K\right\}. \end{array}$$

**Figure 4:**
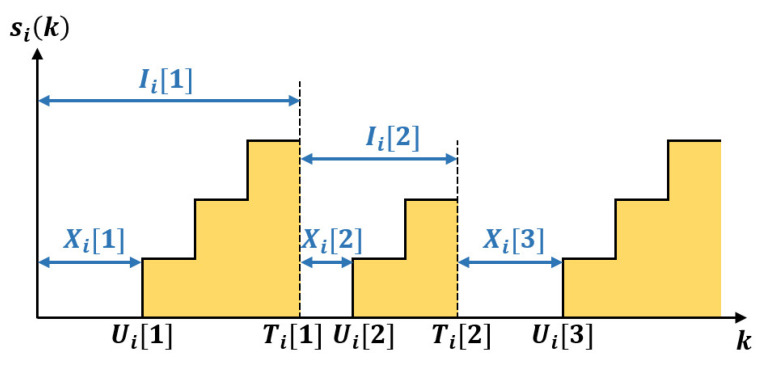
The definition of $I_{i}\left[m\right]$ and $X_{i}\left[m\right]$.

**Proof.**  Let Ω be the sample space associated with the network and ω∈Ω be a certain sample path. Let $D_{i}\left(K\right)=\sum_{k=1}^{K}d_{i}\left(k\right)$ denote the number of updates successfully transmitted from node *i* during the first *K* time slots under a certain sample path ω. Define $U_{i}\left[m\right]$ as the time index of the *m*-th arrival of a new update and $T_{i}\left[m\right]$ as the time index of the *m*-th successful transmission from node *i*, as shown in Figure 4. Then, the inter-delivery time can be computed by

(11) $$I_{i}\left[m\right]=T_{i}\left[m\right]-T_{i}\left[m-1\right],\forall m\in \left\{2,\ldots ,D_{i}\left(K\right)\right\}$$

and $I_{i}\left[1\right]=T_{i}\left[1\right]$. Denote the time from the *m*-th delivery to the next arrival as

(12) $$\begin{array}{c} X_{i}\left[m\right]=\min_{j}\left\{U_{i}\left[j\right]|U_{i}\left[j\right]>T_{i}\left[m-1\right]\right\}-T_{i}\left[m-1\right],\forall m\in \left\{2,\ldots ,D_{i}\left(K\right)\right\} \end{array}$$

and $X_{i}\left[1\right]=U_{i}\left[1\right]$ for simplicity.

Then, the *K* time slots can be decoupled as

(13) $$K=\sum_{m=1}^{D_{i}\left(K\right)}I_{i}\left[m\right]+R_{i},\;\forall i\in \left\{1,\ldots ,M\right\},$$

where $R_{i}$ denotes the rest slots after the $D_{i}\left(K\right)$-th delivery. $X_{i}^{\prime }$ denotes the time from the last delivery to the next arrival or *K*. Since the arrival of updates follows a Bernoulli distribution with the parameter $\lambda_{i}$, $X_{i}\left[m\right]$ and $X_{i}^{\prime }$ follow a geometric distribution with the parameter $\lambda_{i}$, which is independent from $I_{i}\left[m\right]$. During each $I_{i}\left[m\right]$, the AoS increases after $X_{i}\left[m\right]$ slots from 0 to $I_{i}\left[m\right]-X_{i}\left[m\right]$, so the time-average AoS of node *i* is

(14) $$\begin{array}{cc} \frac{1}{K}E\left[\sum_{k=1}^{K}s_{i}\left(k\right)\right] & =\frac{1}{K}E\left[\sum_{m=1}^{D_{i}\left(K\right)}\frac{\left(I_{i}\left[m\right]-X_{i}\left[m\right]\right)\left(I_{i}\left[m\right]-X_{i}\left[m\right]+1\right)}{2}+\frac{\left(R_{i}-X_{i}^{\prime }\right)\left(R_{i}-X_{i}^{\prime }+1\right)}{2}\right] \end{array}$$

(15) $$\begin{array}{cc} \; & \overset{\left(a\right)}{=}\frac{1}{2K}E\left[\sum_{m=1}^{D_{i}\left(K\right)}\left[I_{i}^{2}\left[m\right]+X_{i}^{2}\left[m\right]-X_{i}\left[m\right]\right]+R_{i}^{2}+X_{i}^{\prime 2}-X_{i}^{\prime }-2KX_{i}\left[m\right]+K\right] \end{array}$$

(16) $$\begin{array}{cc} \; & \overset{\left(b\right)}{=}\frac{1}{2K}E\left[\sum_{m=1}^{D_{i}\left(K\right)}I_{i}^{2}\left[m\right]+R_{i}^{2}+\left(D_{i}\left(K\right)+1\right)\left(X_{i}^{\prime 2}-X_{i}^{\prime }\right)-2KX_{i}^{\prime }+K\right], \end{array}$$

where (*a*) is derived by merging linear terms to *K* using Equation (13) and (*b*) is because $X_{i}\left[m\right]$ and $X_{i}^{\prime }$ are independent identically.

The first part of Equation (16) can be lower bounded by Jensen’s inequality

(17) $$\begin{array}{c} \sum_{m=1}^{D_{i}\left(K\right)}I_{i}^{2}\left[m\right]+R_{i}^{2}\geq \frac{1}{D_{i}\left(K\right)+1}{\left[\sum_{m=1}^{D_{i}\left(K\right)}I_{i}\left[m\right]+R_{i}\right]}^{2}=\frac{K^{2}}{D_{i}\left(K\right)+1}. \end{array}$$

To calculate the second part of Equation (16), we can use the property of $X_{i}^{\prime }$ and ${\hat{q}}_{i}$:

(18) $$\begin{array}{cc} E[X_{i}^{\prime }] & =\frac{1}{\lambda_{i}}, \end{array}$$

(19) $$\begin{array}{cc} E[X_{i}^{\prime 2}] & =\frac{2-\lambda_{i}}{\lambda_{i}^{2}}, \end{array}$$

(20) $$\begin{array}{cc} \lim_{K\to \infty }\frac{D_{i}\left(K\right)}{K} & ={\hat{q}}_{i}. \end{array}$$

Combining Equations (16)–(19) and (2), we have

(21) $$\begin{array}{c} \frac{1}{K}E\left[\sum_{k=1}^{K}s_{i}\left(k\right)\right]\geq \frac{K}{2(D_{i}\left(K\right)+1)}+\frac{D_{i}\left(K\right)+1}{K}\cdot \frac{1-\lambda_{i}}{\lambda_{i}^{2}}-\frac{1}{\lambda_{i}}+\frac{1}{2}. \end{array}$$

Take K→∞ in Equation (21) and apply Equation (20):

(22) $$\lim_{K\to \infty }\frac{1}{K}E\left[\sum_{k=1}^{K}s_{i}\left(k\right)\right]\geq \frac{1}{2{\hat{q}}_{i}}+\frac{1-\lambda_{i}}{\lambda_{i}^{2}}{\hat{q}}_{i}-\frac{2-\lambda_{i}}{2\lambda_{i}}.$$

Summing over i∈{1,…,M} gives the limitation of $J_{K}$:

(23) $$\lim_{K\to \infty }J_{K}\geq \frac{1}{2M}\sum_{i=1}^{M}\alpha_{i}\left(\frac{1}{{\hat{q}}_{i}}+\frac{2-2\lambda_{i}}{\lambda_{i}^{2}}{\hat{q}}_{i}-\frac{2-\lambda_{i}}{\lambda_{i}}\right).$$

The lower bound for Equations (10a)–(10c) is proved.    □

## 4. Scheduling Policies

In this section, we propose three scheduling policies. When a central controller schedules the transmissions, we propose the optimal stationary randomized policy in the case where transmission acknowledgement is unused and we propose the max-weight policy in the case where the central controller can get transmission acknowledgement. We consider the BS as the central controller for simplicity. When there is no central scheduling and each node is unaware of the status of the other nodes, we propose the distributed policy.

### 4.1. Optimal Stationary Randomized Policy

In the case without transmission acknowledgement, the BS cannot compute the AoS of the nodes simultaneously. Therefore, the scheduling policy needs to be independent of the AoS. If a policy is entirely based on probability and will not change with the state of the network, it is called a stationary randomized policy and denoted by $R\in \Pi_{R}$. A stationary randomized policy *R* chooses node *i* to transmit with probability $\mu_{i}\in \left(0,1\right]$ and keeps idle with probability $\mu_{idle}$. $\mu_{i}$ has the property that $\sum_{i=1}^{M}\mu_{i}+\mu_{idle}=1$ and $\mu_{i}=E\left[u_{i}\left(k\right)\right]$. Then, we can determine the throughput and AoS of the stationary randomized policy *R*:

**Proposition** **1.** *The long-term throughput and the expected time-average AoS of node i under a policy $R\in \Pi_{R}$ is*

(24) $$\begin{array}{cc} {\hat{q}}_{i}^{R} & =p_{i}\mu_{i}; \end{array}$$

(25) $$\begin{array}{cc} \lim_{K\to \infty }\frac{1}{K}E\left[\sum_{k=1}^{K}s_{i}\left(k\right)\right] & =\frac{1}{p_{i}\mu_{i}}-\frac{1}{\lambda_{i}+p_{i}\mu_{i}-\lambda_{i}p_{i}\mu_{i}}. \end{array}$$

**Proof.**  Since the probabilities of both transmitting to node *i* and transmitting successfully—which refer to $\mu_{i}$ and $p_{i}$, respectively—are determined and independent, the BS receives an update from node *i* in a time slot with probability $p_{i}\mu_{i}$. This implies the long-term throughput ${\hat{q}}_{i}^{R}=p_{i}\mu_{i}$. The inter-delivery time $I_{i}\left[m\right]$ now follows a geometric distribution with parameter $p_{i}\mu_{i}$.

Let the sum AoS during an inter-delivery time $I_{i}\left[m\right]$ be $A_{i}\left[m\right]=\sum_{k=k_{i}\left[m\right]+1}^{k_{i}\left[m\right]+I_{i}\left[m\right]}S_{i}\left(k\right)$, where $k_{i}\left[m\right]=\sum_{j=1}^{m-1}I_{i}\left[j\right]$ if m>1 and $k_{i}\left[m\right]=0$ if m=1. $A_{i}\left[m\right]$ can be derived by summing over the possible values of waiting time $X_{i}\left[m\right]$.

(26) $$\begin{array}{cc} A_{i}\left[m\right]= & \sum_{x=1}^{I_{i}\left[m\right]}\frac{1}{2}\left(I_{i}\left[m\right]-x+1\right)\left(I_{i}\left[m\right]-x\right)\lambda_{i}{\left(1-\lambda_{i}\right)}^{x-1} \end{array}$$

(27) $$\begin{array}{cc} = & \frac{1}{2}I_{i}\left[m\right]\left(I_{i}\left[m\right]+1\right)-\frac{I_{i}\left[m\right]}{\lambda_{i}}+\frac{1-\lambda_{i}}{\lambda_{i}^{2}}\left[1-{\left(1-\lambda_{i}\right)}^{I_{i}\left[m\right]+1}\right]. \end{array}$$

The distribution of $I_{i}\left[m\right]$ gives

(28) $$\begin{array}{cc} E[I_{i}\left[m\right]] & =\frac{1}{p_{i}\mu_{i}}; \end{array}$$

(29) $$\begin{array}{cc} E[I_{i}^{2}\left[m\right]] & =\frac{2-p_{i}\mu_{i}}{{\left(p_{i}\mu_{i}\right)}^{2}}; \end{array}$$

(30) $$\begin{array}{cc} E[{\left(1-\lambda_{i}\right)}^{I_{i}\left[m\right]}] & =\frac{p_{i}\mu_{i}\left(1-\lambda_{i}\right)}{1-\left(1-\lambda_{i}\right)\left(1-p_{i}\mu_{i}\right)}. \end{array}$$

Combining Equations (27)–(30), the expectation of $A_{i}\left[m\right]$ is given as

(31) $$E\left[A_{i}\left[m\right]\right]=\frac{1}{{\left(p_{i}\mu_{i}\right)}^{2}}-\frac{1}{p_{i}\mu_{i}\left(\lambda_{i}+p_{i}\mu_{i}-\lambda_{i}p_{i}\mu_{i}\right)}.$$

The inter-delivery times $I_{i}\left[m\right]$, $\forall m\in \{1,\ldots ,D_{i}\left(K\right)\}$ for each node *i* are independently identically distributed, so the expected time-average AoS of node *i* is equal to the ratio of expectation of $A_{i}\left[m\right]$ to $I_{i}\left[m\right]$, which leads to Equation (25).    □

Substituting Equations (24) and (25) into Equations (8a)–(8c), the computation of the optimal stationary randomized policy can be formulated as follows.

**Problem** **2** (AoS optimization over stationary randomized policies).

(32a) $$AoS_{R^{*}}=\min_{R\in \Pi_{R}}\left\{\frac{1}{M}\sum_{i=1}^{M}\alpha_{i}\left(\frac{1}{p_{i}\mu_{i}}-\frac{1}{\lambda_{i}+p_{i}\mu_{i}-\lambda_{i}p_{i}\mu_{i}}\right)\right\},$$

(32b) $$\begin{array}{cc} s.t.\; & p_{i}\mu_{i}\geq q_{i},\;\forall i\in \left\{1,\ldots ,M\right\}; \end{array}$$

(32c) $$\begin{array}{cc} \; & \sum_{i=1}^{M}\mu_{i}\leq 1. \end{array}$$

Compare Equations (32a)–(32c) with the lower bound of Equations (10a)–(10c). The throughput ${\hat{q}}_{i}^{\pi }$ solving the lower bound can also satisfy Equations (32b) and (32c). Replacing $p_{i}\mu_{i}$ with ${\hat{q}}_{i}^{\pi }$ and subtracting the expression in the minimization function of $AoS_{R^{*}}$ from that of $2AoS_{LB}$ gives

$$\begin{array}{c} \frac{1}{M}\sum_{i=1}^{M}\alpha_{i}\left(\frac{1}{{\hat{q}}_{i}^{\pi }}+\frac{2-2\lambda_{i}}{\lambda_{i}^{2}}{\hat{q}}_{i}^{\pi }-\frac{2-\lambda_{i}}{\lambda_{i}}\right)-\frac{1}{M}\sum_{i=1}^{M}\alpha_{i}\left(\frac{1}{{\hat{q}}_{i}^{\pi }}-\frac{1}{\lambda_{i}+{\hat{q}}_{i}^{\pi }-\lambda_{i}{\hat{q}}_{i}^{\pi }}\right) \end{array}$$

(33) $$\begin{array}{c} =\frac{1}{M}\sum_{i=1}^{M}\alpha_{i}\left(\frac{2-2\lambda_{i}}{\lambda_{i}^{2}}{\hat{q}}_{i}^{\pi }-\frac{2-\lambda_{i}}{\lambda_{i}}+\frac{1}{1-\left(1-\lambda_{i}\right)\left(1-{\hat{q}}_{i}^{\pi }\right)}\right) \end{array}$$

(34) $$\begin{array}{c} >\frac{1}{M}\sum_{i=1}^{M}\alpha_{i}\left(\frac{2-2\lambda_{i}}{\lambda_{i}^{2}}{\hat{q}}_{i}^{\pi }-\frac{2-\lambda_{i}}{\lambda_{i}}+1\right) \end{array}$$

(35) $$\begin{array}{c} =\frac{1}{M}\sum_{i=1}^{M}\alpha_{i}\left(\frac{2\left(1-\lambda_{i}\right)\left({\hat{q}}_{i}^{\pi }-\lambda_{i}\right)}{\lambda_{i}^{2}}\right) \end{array}$$

Though Equation (35) is smaller than 0 for ${\hat{q}}_{i}^{\pi }<\lambda_{i}$, it is much smaller than the common part $1/M\sum_{i=1}^{M}\alpha_{i}/{\hat{q}}_{i}^{\pi }$. Therefore, $AoS_{R^{*}}$ can be bounded by $2AoS_{LB}$ adding a small incremental.

Solving Problem 2 means finding the optimal set of scheduling probabilities ${\left\{\mu_{i}^{*}\right\}}_{i=1}^{M}$. For simplicity, we use $B_{i}\left(\mu_{i}\right)$ to represent $\frac{\alpha_{i}}{M}\left(\frac{1}{p_{i}\mu_{i}}-\frac{1}{\lambda_{i}+p_{i}\mu_{i}-\lambda_{i}p_{i}\mu_{i}}\right)$. Let $\omega_{i}\geq 0$, ∀i∈{1,…,M} and γ≥0 be the Lagrange multiplier. The Lagrange equation is defined as

(36) $$L\left(\mu_{i},\omega_{i},\gamma \right)=\sum_{i=1}^{M}B_{i}\left(\mu_{i}\right)+\sum_{i=1}^{M}\omega_{i}\left(q_{i}-p_{i}\mu_{i}\right)+\gamma (\sum_{i=1}^{M}\mu_{i}-1).$$

Then, the KKT conditions are

(37) $$\begin{array}{cc} \nabla_{\mu_{i}}L\left(\mu_{i},\omega_{i},\gamma \right)=\frac{\partial B_{i}\left(\mu_{i}\right)}{\partial \mu_{i}}-\omega_{i}p_{i}+\gamma & =0,\;\forall i; \end{array}$$

(38) $$\begin{array}{cc} \omega_{i}\left(q_{i}-p_{i}\mu_{i}\right) & =0,\;\forall i; \end{array}$$

(39) $$\begin{array}{cc} \gamma \left(\sum_{i=1}^{M}\mu_{i}-1\right) & =0. \end{array}$$

We change the form of $B_{i}\left(\mu_{i}\right)$ as follows

(40) $$\begin{array}{cc} B_{i}\left(\mu_{i}\right) & =\frac{\alpha_{i}}{M}\left(\frac{1}{p_{i}\mu_{i}}-\frac{1}{\lambda_{i}+p_{i}\mu_{i}-\lambda_{i}p_{i}\mu_{i}}\right) \end{array}$$

(41) $$\begin{array}{cc} \; & =\frac{\alpha_{i}}{Mp_{i}\mu_{i}}\left(1-\frac{1}{\frac{\lambda_{i}}{p_{i}\mu_{i}}+1-\lambda_{i}}\right). \end{array}$$

$B_{i}\left(\mu_{i}\right)$ can be expressed as the product of two parts that are both positive and monotonically decreasing with $\mu_{i}$, so $B_{i}\left(\mu_{i}\right)$ monotonically decreases with $\mu_{i}$ and $\frac{\partial B_{i}\left(\mu_{i}\right)}{\partial \mu_{i}}<0$. This gives

(42) $$\gamma =-\frac{\partial B_{i}\left(\mu_{i}\right)}{\partial \mu_{i}}+\omega_{i}p_{i}>\omega_{i}p_{i}\geq 0.$$

Combining Equations (39) and (42) we get

(43) $$\sum_{i=1}^{M}\mu_{i}=1.$$

According to Equation (38), a node *i* indicates whether $\omega_{i}=0$ or $p_{i}\mu_{i}=q_{i}$. If $p_{i}\mu_{i}=q_{i}$, $\omega_{i}\geq 0$ and $\gamma \geq -\frac{\partial B_{i}\left(\mu_{i}\right)}{\partial \mu_{i}}$. If $\omega_{i}=0$, Equation (42) turns to $\gamma =-\frac{\partial B_{i}\left(\mu_{i}\right)}{\partial \mu_{i}}$. We can derive the monotonicity of $\frac{\partial B_{i}\left(\mu_{i}\right)}{\partial \mu_{i}}$ by calculating the second partial derivative of $B_{i}\left(\mu_{i}\right)$.

(44) $$\begin{array}{c} \frac{\partial^{2}B_{i}\left(\mu_{i}\right)}{\partial \mu_{i}^{2}}=\frac{\alpha_{i}p_{i}^{2}}{M}\left(\frac{1}{{\left(p_{i}\mu_{i}\right)}^{3}}-\frac{{\left(1-\lambda_{i}\right)}^{2}}{{\left(\lambda_{i}+p_{i}\mu_{i}-\lambda_{i}p_{i}\mu_{i}\right)}^{3}}\right). \end{array}$$

It is clear that $1>{\left(1-\lambda_{i}\right)}^{2}$ and ${\left(p_{i}\mu_{i}\right)}^{3}<{\left(\lambda_{i}+p_{i}\mu_{i}-\lambda_{i}p_{i}\mu_{i}\right)}^{3}$, so $\frac{\partial^{2}B_{i}\left(\mu_{i}\right)}{\partial \mu_{i}^{2}}>0$ and $\frac{\partial B_{i}\left(\mu_{i}\right)}{\partial \mu_{i}}$ increases monotonically with $\mu_{i}$.

Note that γ is shared by all $\mu_{i}$, so $\mu_{i}$ can be derive by comparing $-\frac{\partial B_{i}\left(q_{i}/p_{i}\right)}{\partial \mu_{i}}$ and γ. When $-\frac{\partial B_{i}\left(q_{i}/p_{i}\right)}{\partial \mu_{i}}>\gamma$, $\mu_{i}$ is the solution of $\gamma =-\frac{\partial B_{i}\left(\mu_{i}\right)}{\partial \mu_{i}}$, which is bigger than $\frac{q_{i}}{p_{i}}$; otherwise, it is $\mu_{i}=\frac{q_{i}}{p_{i}}$.

We can get the optimal ${\left\{\mu_{i}^{*}\right\}}_{i=1}^{M}$ with the following steps. Start from a large γ so that $\mu_{i}=\frac{q_{i}}{p_{i}}$, ∀i. Now, we have $\sum_{i=1}^{M}\mu_{i}=\sum_{i=1}^{M}\frac{q_{i}}{p_{i}}\leq 1$. When γ decreases to the point that $\gamma <-\frac{\partial B_{i}\left(q_{i}/p_{i}\right)}{\partial \mu_{i}}$, $\mu_{i}$ will be replaced by the solution of $\gamma =-\frac{\partial B_{i}\left(\mu_{i}\right)}{\partial \mu_{i}}$, which increases gradually with the decrease in γ. Let γ decrease until $\sum_{i=1}^{M}\mu_{i}$ reaches 1. That is when the solution is reached.

**Theorem** **2** (Optimal stationary randomized policy). The optimal stationary randomized policy $R^{*}$ solving Equations (32a)–(32c) is the set of scheduling probabilities ${\left\{\mu_{i}^{*}\right\}}_{i=1}^{M}$ derived from Algorithm 1.

**Algorithm 1** Solution to the optimal stationary randomized policy
1:**Initialization: ***M*, $\alpha_{i}$, $p_{i}$, $q_{i}$, $\lambda_{i}$ and $B_{i}\left(\mu_{i}\right)=\frac{\alpha_{i}}{M}\left(\frac{1}{p_{i}\mu_{i}}-\frac{1}{\lambda_{i}+p_{i}\mu_{i}-\lambda_{i}p_{i}\mu_{i}}\right)$2:$\mu_{i}=\frac{q_{i}}{p_{i}}$, ∀i∈{1,2,…,M}3:

$\gamma_{i}=-\frac{\partial B_{i}\left(\mu_{i}\right)}{\partial \mu_{i}}$

4:

$\gamma =\max_{i}\gamma_{i}$

5:
**while** $\sum_{i=1}^{M}\mu_{i}<1$ **do**
6:   decrease γ slightly7:   **for ** each i∈{1,2,…,M} **do**8:     **if ** $-\frac{\partial B_{i}\left(\mu_{i}\right)}{\partial \mu_{i}}>\gamma$ **then**9:        replace $\mu_{i}$ by the solution of $\gamma =-\frac{\partial B_{i}\left(\mu_{i}\right)}{\partial \mu_{i}}$10:     **end if**11:   **end for**12:
**end while**
13:**Output: **$\gamma^{*}=\gamma$ and $\mu_{i}^{*}=\mu_{i}$, ∀i∈{1,2,…,M}


### 4.2. Max-Weight Policy

When the transmission acknowledgement is used, the BS can make decisions according to the AoS of the network. The idea of the max-weight policy is to optimize the network by controlling the expected increase in the Lyapunov function. The Lyapunov function becomes large with a large AoS or a lack in throughput.

We define the throughput debt $x_{i}\left(k\right)$ as the difference between the target throughput and the number of actually delivered updates. A large $x_{i}\left(k\right)$ indicates that node *i* needs more transmissions to satisfy the throughput constraint.

(45) $$x_{i}\left(k+1\right)=kq_{i}-\sum_{t=1}^{k}d_{i}\left(t\right).$$

If $x_{i}\left(k\right)<0$, node *i* leads the target throughput so that the BS has no motivation to choose node *i* from the perspective of throughput. Therefore, we just focus on positive $x_{i}\left(k\right)$. We use operator ${\left(\cdot \right)}^{+}=\max\left\{\left(\cdot \right),0\right\}$ to denote the positive part of the throughput debt $x_{i}^{+}\left(k\right)=\max\left\{x_{i}\left(k\right),0\right\}$. Then, the throughput constraint can be satisfied when

(46) $$\lim_{K\to \infty }\frac{1}{K}E\left[\sum_{k=1}^{K}x_{i}^{+}\left(k\right)\right]<\infty .$$

Define the system state at the beginning of time slot *k* as $S_{k}={\left(s_{i}\left(k\right),x_{i}^{+}\left(k\right)\right)}_{i=1}^{M}$ and the Lyapunov function as

(47) $$L\left(S_{k}\right):=\frac{1}{2}\sum_{i=1}^{M}\left(\alpha_{i}s_{i}^{2}\left(k\right)+V{\left(x_{i}^{+}\left(k\right)\right)}^{2}\right),$$

where *V* is a positive real number indicating the importance of the throughput constraint. Thus, $L(S_{k})$ grows with the AoS or the throughput debt. We define the Lyapunov drift as

(48) $$\Delta \left(S_{k}\right):=E\left\{L\left(S_{k+1}\right)-L\left(S_{k}\right)|S_{k}\right\}.$$

The Lyapunov drift describes the expected change in the Lyapunov function in each time slot, so $L(S_{k})$ can be restricted by reducing $\Delta (S_{k})$. To help execute the max-weight policy, an upper bound of $\Delta (S_{k})$ is given below.

**Proposition** **2.** *The Lyapunov drift can be upper-bounded by*

(49) $$\Delta \left(S_{k}\right)\leq -\sum_{i=1}^{M}W_{i}\left(k\right)E\left\{u_{i}\left(k\right)|S_{k}\right\}+C\left(k\right),$$

*where*

(50) $$\begin{array}{cc} W_{i}\left(k\right) & =\frac{\alpha_{i}p_{i}}{2}{\left(s_{i}\left(k\right)+1\right)}^{2}+Vp_{i}x_{i}^{+}\left(k\right); \end{array}$$

(51) $$\begin{array}{cc} C(k) & =\sum_{i=1}^{M}\left(\alpha_{i}\left(s_{i}\left(k\right)+\frac{1}{2}\right)+V\left(q_{i}x_{i}^{+}\left(k\right)+\frac{1}{2}\right)\right). \end{array}$$

**Proof.** 
(52) $$\begin{array}{cc} \Delta (S_{k}) & =\frac{1}{2}\sum_{i=1}^{M}\alpha_{i}E\left\{s_{i}^{2}\left(k+1\right)-s_{i}^{2}\left(k\right)|S_{k}\right\}\\ \; & +\frac{V}{2}\sum_{i=1}^{M}E\left\{{\left[x_{i}^{+}\left(k+1\right)\right]}^{2}-{\left[x_{i}^{+}\left(k\right)\right]}^{2}|S_{k}\right\}. \end{array}$$

Consider the latter part first. By the definition of $x_{i}\left(k\right)$,

(53) $$\begin{array}{cc} {\left[x_{i}^{+}\left(k+1\right)\right]}^{2} & ={\left[\max\left\{x_{i}\left(k\right)-d_{i}\left(k\right)+q_{i},0\right\}\right]}^{2}\\ \; & \leq {\left[\max\left\{x_{i}^{+}\left(k\right)-d_{i}\left(k\right)+q_{i},0\right\}\right]}^{2}\\ \; & \leq {\left[x_{i}^{+}\left(k\right)-d_{i}\left(k\right)+q_{i}\right]}^{2}\\ \; & \leq {\left[x_{i}^{+}\left(k\right)\right]}^{2}+2x_{i}^{2}\left(k\right)\left(-d_{i}\left(k\right)+q_{i}\right)+1. \end{array}$$

Take the expectation and use the property of $d_{i}\left(k\right)$:

(54) $$\begin{array}{c} \frac{V}{2}\sum_{i=1}^{M}E\left\{{\left[x_{i}^{+}\left(k+1\right)\right]}^{2}-{\left[x_{i}^{+}\left(k\right)\right]}^{2}|S_{k}\right\}\leq V\sum_{i=1}^{M}\left(x_{i}^{+}\left(k\right)\left(-p_{i}E\left\{u_{i}\left(k\right)|S_{k}\right\}+q_{i}\right)+\frac{1}{2}\right). \end{array}$$

The next step is dealing with the former part of $\Delta (S_{k})$.

(55) $$\begin{array}{cc} E\{s_{i}^{2}\left(k+1\right)\} & \leq 0\cdot E\left\{d_{i}\left(k\right)\right\}+{\left(s_{i}\left(k\right)+1\right)}^{2}\left(1-E\left\{d_{i}\left(k\right)\right\}\right) \end{array}$$

(56) $$\begin{array}{cc} \; & ={\left(s_{i}\left(k\right)+1\right)}^{2}\left(1-p_{i}E\left\{u_{i}\left(k\right)|S_{k}\right\}\right). \end{array}$$

The inequality is because the AoS remains 0 rather than $s_{i}\left(k\right)+1$ if $s_{i}\left(k\right)=0$. So, we have

(57) $$\begin{array}{c} E\left\{s_{i}^{2}\left(k+1\right)-s_{i}^{2}\left(k\right)|S_{k}\right\}\leq -{\left(s_{i}\left(k\right)+1\right)}^{2}p_{i}E\left\{u_{i}\left(k\right)|S_{k}\right\}+2s_{i}\left(k\right)+1. \end{array}$$

Combining Equations (52), (54), and (57) completes the proof.    □

We can find from the form of Equations (49)–(51) that $W_{i}\left(k\right)$ and C(k) fully depend on the system state S(k). What our policy controls is the set of $u_{i}\left(k\right)$. We want to choose a node *i* that can reduce $S_{k}$ most to transmit in each time slot. So, the node with the largest $W_{i}\left(k\right)$ is the optimal choice. That is how max-weight works.

**Lemma** **1.** *The max-weight policy satisfies any feasible set of minimum throughput requirements ${\left\{q_{i}\right\}}_{i=1}^{M}$.*

**Lemma** **2** (Upper bound of the max-weight policy). *The optimal expected time-average AoS of the max-weight policy can be upper-bounded by*

(58) $$AoS_{MW}\leq \frac{2}{M}\sum_{i=1}^{M}\frac{\alpha_{i}}{p_{i}\mu_{i}}+V.$$

Lemmas 1 and 2 are from Theorems 6 and 7 in [25]. The upper bound of max-weight is near four times the lower bound in Equations (10a)–(10c). Though $AoS_{MW}$ has a large upper bound compared to $AoS_{R^{*}}$, it was found in the numerical simulation that max-weight performs much better than the optimal stationary randomized policy.

### 4.3. Distributed Policy

When the number of nodes increases, it is difficult and costly to apply the centralized policies proposed above. As an alternative, we study the distributed policy, where a node knows neither the system states of the other nodes nor when the other nodes are transmitting. It is possible that more than one node transmits to the BS in the same time slot. So, we first relax the bandwidth constraints (Equation (8c)) to a time-average constraint

(59) $$\frac{1}{K}\sum_{k=1}^{K}\sum_{i=1}^{M}E\left[u_{i}\left(k\right)\right]\leq 1.$$

Then, we solve the optimization problem with the Lagrange multiplier method.

**Problem** **3** (Relaxed AoS optimization).

(60a) $$\begin{array}{c} AoS_{re}^{*}=\min_{\pi \in \Pi }\left\{\lim_{K\to \infty }\frac{1}{KM}\sum_{k=1}^{K}\sum_{i=1}^{M}\left[\alpha_{i}E\left[s_{i}\left(k\right)\right]+C\left(E\left[u_{i}\left(k\right)\right]-\frac{1}{M}\right)+\theta_{i}\left(\frac{q_{i}}{p_{i}}-E\left[u_{i}\left(k\right)\right]\right)\right]\right\}, \end{array}$$

(60b) $$\begin{array}{cc} s.t.\; & \theta_{i}\geq 0,\;\forall i\in \left\{1,\ldots ,M\right\}; \end{array}$$

(60c) $$\begin{array}{cc} \; & C\geq 0, \end{array}$$

where $\theta_{i}$ and *C* are Lagrange multipliers respectively associated with throughput constraints $E\left[u_{i}\left(k\right)\right]\geq \frac{q_{i}}{p_{i}}$ and the relaxed bandwidth constraint (Equation (59)).

The form of Equation (60a) is independent for each node. Thus, the relaxed optimization problems associated with each individual node can be solved separately. From the perspective of the nodes, each node only considers its own AoS, and the action space is simplified to either transmitting or not in each time slot. As the following proposition shows, the transmission policy has a threshold structure.

**Proposition** **3.** (Threshold policy). *The relaxed optimization problem (Equations* (60a)–(60c)) *for each single node i has an optimal threshold policy. Under the policy, node i chooses to transmit when $s_{i}\left(k\right)\geq S_{i}$ and to remain idle when $0\leq s_{i}\left(k\right)<S_{i}$ in time slot k. For given C and $\theta_{i}$, ∀i∈{1,…,M}, and if $C>\theta_{i}$, the threshold is*

(61) $$\begin{array}{c} S_{i}=\left\lfloor \frac{5}{2}-\frac{1}{p_{i}}-\frac{1}{\lambda_{i}}+\sqrt{{\left(\frac{5}{2}-\frac{1}{p_{i}}-\frac{1}{\lambda_{i}}\right)}^{2}+2\left(\frac{C-\theta_{i}}{\alpha_{i}p_{i}}-\frac{1-2\lambda_{i}}{\lambda_{i}}\frac{1-p_{i}}{p_{i}}\right)}\right\rfloor . \end{array}$$

*If $C\leq \theta_{i}$, $S_{i}=0$.*

When $C\leq \theta_{i}$, the coefficient of $E\left[u_{i}\left(k\right)\right]$ in the objective function (Equation (60a)) is $C-\theta_{i}\leq 0$. In that case, node *i* will always choose to transmit, as there is no constraint on transmission, which is equivalent to $S_{i}=0$. When $C>\theta_{i}$, the proof of Proposition 3 follows from Appendix E in [19].

The final issue is how to choose the values of *C* and $\theta_{i}$. In order to examine the influence of the multipliers on the objective function, we can derive a lower bound of $AoS_{re}^{*}$. Let $C>\theta_{i}$, as the function will fail to meet the bandwidth constraint otherwise. We use $E\left[s_{i}\left(k\right)\right]\geq S_{i}-1$ and $E\left[u_{i}\left(k\right)\right]\geq 0$

(62) $$\begin{array}{c} \max_{C,\theta_{i}}\left(L\left(C,\theta_{i}\right)\right)\leq AoS_{re}^{*}, \end{array}$$

where

(63) $$\begin{array}{c} L\left(C,\theta_{i}\right)=\frac{1}{M}\sum_{i=1}^{M}\left[\alpha_{i}\left(S_{i}-1\right)-\frac{C}{M}+\frac{q_{i}\theta_{i}}{p_{i}}\right]. \end{array}$$

Taking the partial derivative of *C* and $\theta_{i}$

(64) $$\begin{array}{cc} \frac{\partial L(C,\theta_{i})}{\partial \theta_{i}} & =-\frac{1}{M\phi_{i}}+\frac{q_{i}}{Mp_{i}}; \end{array}$$

(65) $$\begin{array}{cc} \frac{\partial L(C,\theta_{i})}{\partial C} & =\frac{1}{M}\left(-1+\sum_{i=1}^{M}\frac{1}{\phi_{i}}\right), \end{array}$$

where

(66) $$\begin{array}{c} \phi_{i}=p_{i}\sqrt{{\left(\frac{5}{2}-\frac{1}{p_{i}}-\frac{1}{\lambda_{i}}\right)}^{2}+2\left(\frac{C-\theta_{i}}{\alpha_{i}p_{i}}-\frac{1-2\lambda_{i}}{\lambda_{i}}\frac{1-p_{i}}{p_{i}}\right)}. \end{array}$$

Let Equation (64) equal 0

(67) $$\begin{array}{cc} \frac{1}{\phi_{i}} & =\frac{q_{i}}{p_{i}},\;\forall i\in \left\{1,\ldots ,M\right\}; \end{array}$$

Substituting Equation (67) into (65), we derive

(68) $$\frac{\partial L(C,\theta_{i})}{\partial C}=\frac{1}{M}\left(-1+\sum_{i=1}^{M}\frac{q_{i}}{p_{i}}\right)\leq 0.$$

To maximize $L(C,\theta_{i})$, we can continuously decrease *C* until Equation (65) equals 0. The initial value of *C* is given by Equation (67) and $C-\theta_{i}\geq 0$, ∀i. The values of *C* and $\theta_{i}$ can be given by Algorithm 2.

Then, the distributed policy works in the following way. Each node *i* monitors its own AoS $s_{i}\left(k\right)$ and transmits to the BS when $s_{i}\left(k\right)$ exceeds the threshold $S_{i}$. If multiple nodes attempt to transmit to the BS in a time slot, only one randomly selected node will be able to transmit, and the transmissions of the other nodes will be treated as failures.
**Algorithm 2 **Solution of the distributed policy1:**Initialization: ***M*, $\alpha_{i}$, $p_{i}$, $q_{i}$ and $\lambda_{i}$2:$\chi_{i}=\frac{\alpha_{i}p_{i}}{2}\left({\left(\frac{1}{q_{i}}\right)}^{2}-{\left(\frac{5}{2}-\frac{1}{p_{i}}-\frac{1}{\lambda_{i}}\right)}^{2}+\frac{2\left(1-2\lambda_{i}\right)\left(1-p_{i}\right)}{\lambda_{i}p_{i}}\right)$, ∀i∈{1,2,…,M}3:$C=\max_{i}\left\{\chi_{i}\right\}$4:$\chi_{i}=\min\left\{C,\chi_{i}\right\}$, ∀i∈{1,2,…,M}5:$\phi_{i}=p_{i}\sqrt{{\left(\frac{5}{2}-\frac{1}{p_{i}}-\frac{1}{\lambda_{i}}\right)}^{2}+2\left(\frac{\chi_{i}}{\alpha_{i}p_{i}}-\frac{\left(1-2\lambda_{i}\right)\left(1-p_{i}\right)}{\lambda_{i}p_{i}}\right)}$, ∀i∈{1,2,…,M}6:**while **$\sum_{i=1}^{M}\phi_{i}^{-1}<1$**do**7:   decrease *C* slightly8:   repeat steps 4 and 59:**end while**10:**Output: **$C^{*}=C$ and $\theta_{i}^{*}=C^{*}-\chi_{i}$, ∀i∈{1,2,…,M}

## 5. Simulation Results

In this section, we provide simulation results for the optimal stationary randomized policy, the max-weight policy, and the distributed policy. For comparison, we simulated the round robin policy, the largest-weighted-debt-first policy, and the drift-plus-penalty policy. The round robin policy, which has no randomness and functions regardless of the system state, chooses each node that will transmit in turn. The largest-weighted-debt-first policy is proposed in [26] and chooses the nodes with the highest $x_{i}\left(k\right)/p_{i}$ in time slot *k*. The drift-plus-penalty policy proposed from [25] chooses the node with the highest $W_{i}\left(k\right)=\frac{\beta_{i}p_{i}}{2}h_{i}\left(k\right)+Vp_{i}x_{i}^{+}\left(k\right)$, where $h_{i}\left(k\right)$ is the age of information of node *i* and $\beta_{i}$ is a positive real value associated with node *i*. When transmission feedback is unavailable, only the optimal stationary randomized policy and the round robin policy can be used.

We simulated a network with *M* nodes. Node *i* has the weight $\alpha_{i}=i/\sum_{i=1}^{M}i$. Arriving rates of the *M* new updates are evenly distributed between 0.5 and 0.1. The set of channel reliability values $p_{i}$ is a vector of *M* evenly spaced points between 0.9 and 0.1. A parameter ε∈[0,1) shows the hardness of the throughput constraints to be satisfied. The set of $q_{i}/p_{i}$ sums to ε and is proportional to a set of *M* evenly spaced points between 0.06 and 0.1. The importance of the throughput constraint in the max-weight was set to V=100.

**Figure 5:**
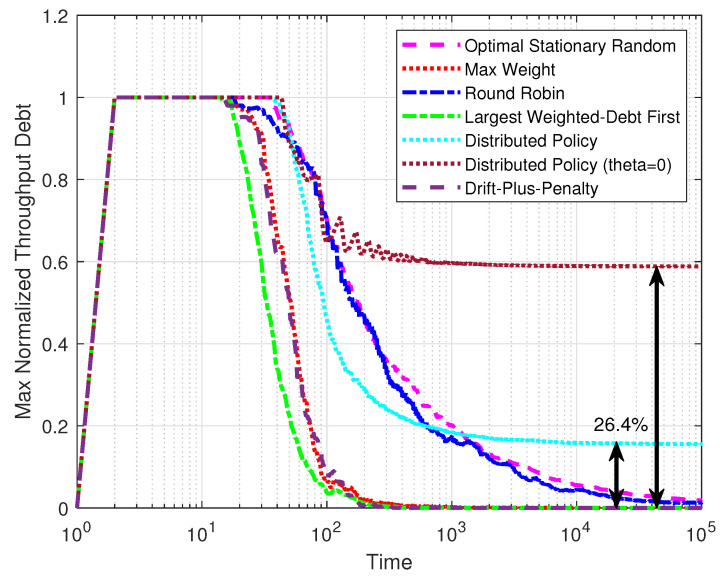
The max normalized throughput debt varied with time.

Figure 5 shows the performance of the throughput of the policies over time. The max normalized throughput debt was defined as $\max_{i}\left\{x_{i}^{+}\left(K+1\right)/\left(Kq_{i}\right)\right\}$. The system was set with M=10, $K=10^{5}$, and ε=0.8, and the results were taken as the average from 100 simulations. After the initial rise, the max normalized throughput debt of the optimal stationary randomized policy, the max-weight policy, the round robin policy, the largest-weighted-debt-first policy, and the drift-plus-penalty policy gradually approached zero with different speeds, which proves that these policies can satisfy throughput constraints in steady state. It is reasonable that the largest-weighted-debt-first policy descended fastest, as its optimization objective is the same as this measurement. Notice that the max normalized throughput debt of the distributed policy could not reach zero, meaning that the distributed policy could not give a throughput guarantee. However, compared with the results for the distributed policy without the multipliers $\theta_{i}$, our distributed policy has made great progress in meeting the throughput constraints.

**Figure 6:**
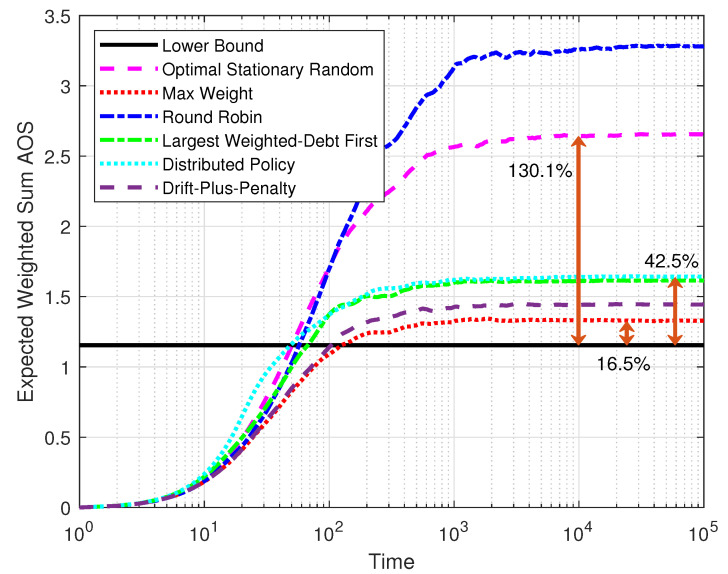
The expected weighted-sum AoS varied with time.

Figure 6 compares the expected weighted-sum AoS of five policies over time and the lower bound defined by Equations (10a)–(10c). The system was set with M=10, $K=10^{5}$, and ε=0.8. The results were taken as the average from 100 simulations. The optimal stationary randomized policy and the round robin policy had significant disadvantages compared to the other policies because they are unable to use the system states. However, the optimal stationary randomized policy performed much better than the round robin policy. The steady AoS of the optimal stationary randomized policy was close to twice the lower bound, which matches the previous analysis. When transmission feedback was available, it was clear that max-weight performed best regarding the expected weighted-sum AoS and was close to the lower bound. Though the AoS of the distributed policy grew faster at the beginning due to the threshold structure, it gradually converged to a good level close to the largest-weighted-debt-first policy.

**Figure 7:**
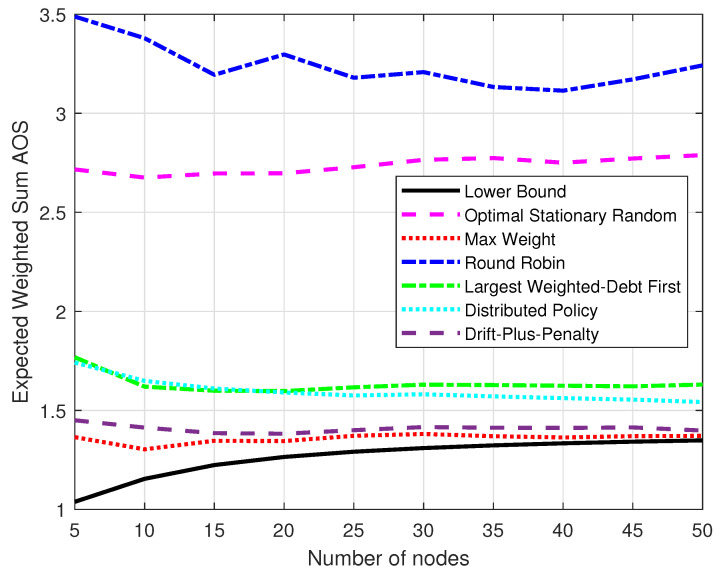
The expected weighted-sum AoS varied with the number of nodes.

Figure 7 compares the expected weighted-sum AoS of the policies and the lower bound with different numbers of nodes, which were set by M∈{5,10,…,45,50}. The total simulation time was $K=10^{5}$. Each result was the average of the data for the time duration $0.9\times 10^{5}$ to $10^{5}$. The hardness of throughput constraints was ε=0.8. The performance of the six policies generally kept stable gaps and moved closer to the lower bound while the number of nodes grew. When the number of nodes was very large, the distributed policy showed greater potential for progress and the AoS of the max-weight policy was very close to the lower bound.

**Figure 8:**
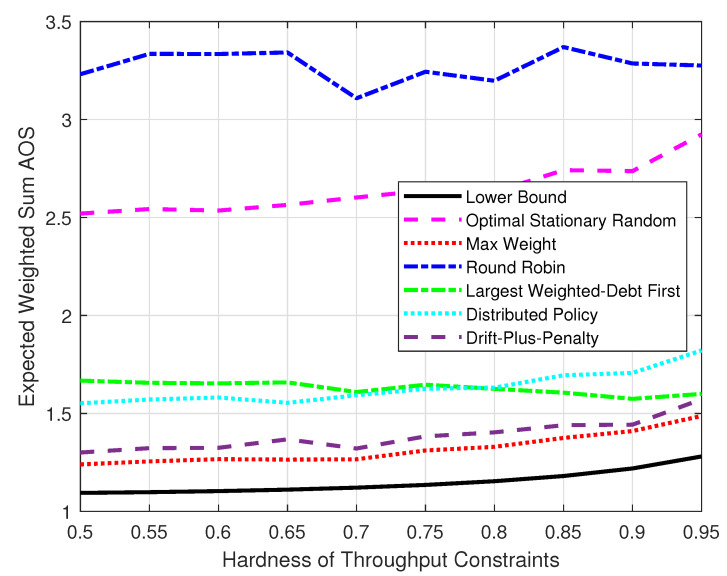
The expected weighted-sum AoS varied with the hardness of the throughput constraints.

Figure 8 compares the expected weighted-sum AoS under different throughput constraints. We set the hardness of the throughput constraints ε to change from 0.5 to 0.95. A larger ε means the throughput constraints are closer to the upper bound limited by Assumption 1. The system was set with M=10, $K=10^{5}$. Each result was the average of the data for the time duration $0.9\times 10^{5}$ to $10^{5}$. When the hardness of satisfying the throughput constraints increased, each policy had to prioritize the throughput and had fewer resources available to reduce the AoS. Thus, it can be seen that the gap between the max-weight, the drift-plus-penalty, and the largest-weighted-debt-first policies and the gap between the optimal stationary randomized and round robin policies decreased with the growth in ε. The distributed policy showed a similar trend as the max-weight policy.

## 6. Conclusions

In this study, we investigated a wireless network that transmits time-sensitive data from multiple nodes to a single base station (BS) over a shared imperfect channel while ensuring the minimum throughput requirement of each node. We measured the freshness of information stored at the BS with a metric called the age of synchronization (AoS). The objective of this study was to minimize the time-average AoS of the entire network while meeting the throughput requirement of each node. We proposed three low-complexity scheduling policies under different scenarios: the optimal stationary randomized policy, the max-weight policy, and the distributed policy. Numerical simulations showed that, in the central control scenario, the optimal stationary randomized policy and the max-weight policy could meet the minimum throughput requirement well. In the case where transmission feedback was unavailable to the BS, the optimal stationary randomized policy showed good AoS performance, and when transmission feedback was available, the max-weight policy closely approximated the AoS lower bound. In the distributed scenario, though the throughput requirement could not be guaranteed, the distributed policy made a great contribution toward meeting the throughput requirement and performed well in terms of AoS.

## Data Availability

The data presented in this study are available on request from the corresponding author. The data are not publicly available due to privacy.
